# Charge-tuneable biexciton complexes in monolayer WSe_2_

**DOI:** 10.1038/s41467-018-05632-4

**Published:** 2018-09-13

**Authors:** Matteo Barbone, Alejandro R.-P. Montblanch, Dhiren M. Kara, Carmen Palacios-Berraquero, Alisson R. Cadore, Domenico De Fazio, Benjamin Pingault, Elaheh Mostaani, Han Li, Bin Chen, Kenji Watanabe, Takashi Taniguchi, Sefaattin Tongay, Gang Wang, Andrea C. Ferrari, Mete Atatüre

**Affiliations:** 1 Cavendish Laboratory, University of Cambridge, JJ Thomson Ave., Cambridge, CB3 0HE UK; 2 Cambridge Graphene Centre, University of Cambridge, Cambridge, CB3 0FA UK; 3 School for Engineering of Matter, Transport and Energy, Arizona State University, Tempe, AZ 85287 USA; 4 National Institute for Materials Science, Tsukuba, Ibaraki 305-0034 Japan

## Abstract

Monolayer transition metal dichalcogenides have strong Coulomb-mediated many-body interactions. Theoretical studies have predicted the existence of numerous multi-particle excitonic states. Two-particle excitons and three-particle trions have been identified by their optical signatures. However, more complex states such as biexcitons have been elusive due to limited spectral quality of the optical emission. Here, we report direct evidence of two biexciton complexes in monolayer tungsten diselenide: the four-particle neutral biexciton and the five-particle negatively charged biexciton. We distinguish these states by power-dependent photoluminescence and demonstrate full electrical switching between them. We determine the band states of the elementary particles comprising the biexcitons through magneto-optical spectroscopy. We also resolve a splitting of 2.5 meV for the neutral biexciton, which we attribute to the fine structure, providing reference for subsequent studies. Our results unveil the nature of multi-exciton complexes in transitionmetal dichalcogenides and offer direct routes towards deterministic control in many-body quantum phenomena.

## Abstract (web summary)

Multi-exciton states may emerge in atomically thin transition metal dichalcogenides as a result of strong many-body interactions. Here, the authors report experimental evidence of four- and five-particle biexciton complexes in monolayer WSe_2_ and their electrical control.

## Introduction

In monolayer (1L) transitionmetal dichalcogenides (TMDs), the three-atom thickness of the material reduces the dielectric screening with respect to their bulk counterparts^1,2^. As a result of this and of their large effective mass, excitons (quasi-particle states formed of electrons and holes via Coulomb interaction) have binding energies of hundreds of meV^1,2^ and are stable at room temperature. The physics of light–matter interaction is also enriched by two inequivalent valleys having opposite spin-locked valley indices^3^ at the K points of the Brillouin zone, in which radiative recombination generates photons carrying opposite angular momenta^4,5^. These properties motivated the exploration of exciton and polariton^6^ condensation^7,8^ and superfluidity^9^, and the exploitation of the spin and valley degrees of freedom as means to carry and manipulate information in quantum optoelectronic devices^3,10^. In the limit of quantum-confined excitons, the presence of localised single-photon emitters that can be induced deterministically^11,12^ and generated by electroluminescence^13^, makes TMDs a promising platform for the field of quantum photonics. Contrary to the exciton^14,15^ and trion^16,17^ states, optical studies of biexciton complexes^18,19^ in 1L-TMDs have been challenging^20–26^: inhomogeneous broadening^27^ and defect bands^28^ have limited their unambiguous identification and control. As a consequence, previous experimental findings^20–23,25,26^ assigned neutral biexcitons a larger binding energy than trions, in contrast to theoretical predictions^29–33^, whereas ref. ^24^ observed a peak in 1L-molybdenum diselenide (MoSe_2_) in the expected energy range, which they labelled as the neutral biexciton.

Here, we use continuous wave photoluminescence (PL) measurements at cryogenic temperature combined with electrical gating and magnetic field to identify the four-particle neutral biexciton (XX^0^) and the five-particle negatively charged biexciton, the quinton^29^ (XX^−^) in 1L-tungsten diselenide (WSe_2_). We also observe a splitting in XX^0^, which we attribute to its fine structure. Our results demonstrate tuneable access to multi-exciton complexes in TMDs and provide new ways to study and control multi-exciton phenomena.

## Results

### Design and optical characterisation of heterostructures

**Fig. 1:**
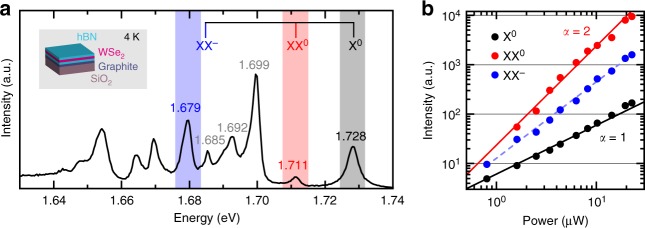
PL spectrum and power dependence of encapsulated 1L-WSe_2_ at 4 K. **a** PL spectrum (black curve, linear scale) of encapsulated 1L-WSe_2_. Excitation wavelength: 658 nm. The top part of the figure lists the calculated spectral locations of X^0^ (grey), XX^0^ (red) and XX^−^ (blue) in the presence of a screening environment. **b** Double logarithmic plot of PL intensity as a function of excitation power for X^0^ (black filled circles), XX^0^ (red filled circles) and XX^−^(blue filled circles). The solid curves represent *I* ∝ *P*^*α*^ for a quadratic (α = 2, red) and linear (α = 1, black) behaviour. The dashed blue curve is a fit to PL intensity, yielding an α of 1.55. For clarity of display, we multiply XX^0^ by 4 and X^0^ by 0.4

We use recent advances in material and device processing^27,34^ to suppress the effects that degrade the optical quality of 1L-WSe_2_. To reduce the PL spectral linewidths^27^ we place a layered material heterostructure (LMH) formed of 1L-WSe_2_ encapsulated between two flakes of multilayer hexagonal boron nitride (ML-hBN) on a Si/SiO_2_ substrate. To suppress the effect of SiO_2_ charge traps we place a few-layer graphene (FLG) crystal below the bottom ML-hBN. The inset of Fig. 1a shows a schematic of the LMH (see Methods, and Supplementary Notes 1 and 2 for sample preparation and characterisation).

We illuminate the LMH with continuous laser excitation at 658 nm and collect its optical emission at 4 K (see Methods for further details on the optical measurements): Fig. 1a is a representative PL spectrum. Consistent with previous reports, we identify the bright neutral exciton^10^, X^0^, at ~1.728 eV (width ~5 meV), the negatively charged intervalley trion^35^, X^−^_inter_, at ~1.699 eV, the negatively charged intravalley trion^35^, X^−^_intra_, at ~1.692 eV, and the dark neutral exciton^36,37^, X^0^_dark_, at ~1.685 eV. Here, *bright* refers to excitons with in-plane dipole and spin-allowed radiative recombination^2,36,37^, whereas *dark* refers to excitons with out-of-plane dipole and spin-forbidden radiative recombination^2,36,37^, for which emission only occurs in plane but is captured partially by our high numerical aperture objective. The peak at ~1.711 eV, ~4 meV wide, is a good candidate for XX^0^, as it appears in the theoretically predicted energy range^29–32^. The peak at ~1.679 eV, ~6 meV wide, was previously labelled as neutral biexciton emission^20^, although it appears in the energy range predicted^29,31^ for XX^−^. In the top part of Fig. 1a, we include the emission energies of single- and multi-exciton species in ML-WSe_2_ calculated via diffusion Quantum Monte Carlo^29^ combined with environment screening (See Methods for details).

### Unveiling the presence and nature of biexcitons

Figure 1b displays the PL intensity *I*, defined as peak area, as a function of excitation power *P* (with *I* ∝ *P*^*α*^) for X^0^ (filled black circles), XX^0^ (filled red circles) and XX^−^ (filled blue circles). For reference, we plot solid curves corresponding to a linear (*α* = 1, black) and quadratic (*α* = 2, red) behaviour. We expect superlinear behaviour for biexcitons reaching *α* = 2 in thermodynamic equilibrium^18,19^. The power dependence of XX^0^ follows the quadratic curve, while that of XX^−^is superlinear with fitted *α* ~ 1.55 ± 0.03 (dashed blue curve). Both trends of XX^0^ and XX^−^are therefore consistent with a biexcitonic origin and contrast the linear behaviour of X^0^. The deviation of XX^−^ from *α* = 2 possibly stems from the competition of electron capture from other optically induced excitons. Remaining peaks of Fig. 1a follow an approximately linear power dependence.

**Fig. 2:**
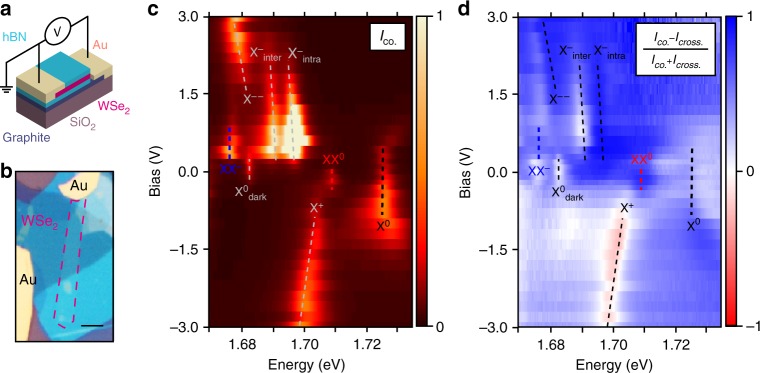
Charge dependence of PL. **a** Schematic and **b** optical image of the charge-tuneable device. The red dashed frame highlights the 1L-WSe_2_ flake. The scale bar is 5 μm. **c** Circular co-polarised PL intensity (*I*_*σ+/σ+*_ *+* *I*_*σ-/σ-*_) as a function of applied bias. The dashed lines are a guide to the eye to highlight each peak. **d** DoP of PL as a function of bias and energy in the same range as (**c**). The colour code is such that blue regions indicate co-polarisation, whereas the red regions indicate counter-polarisation with respect to excitation polarisation

To differentiate the charged and neutral biexciton XX^0^ and XX^−^, we fabricate a charge-tuneable device starting from a new LMH analogous to the first one but with the addition of one electrode to the FLG and of a second electrode to an uncovered 1L-WSe_2_ portion (see Methods). Figure 2a, b shows the schematic and the optical image of the device, respectively. Figure 2c displays how the PL spectrum is modified as a function of voltage *V*. The charging regime modifies the optical signatures of 1L-WSe_2_ strongly. The presence of X^0^ and X^0^_dark_ at *V* ~ 0 V shows that the material has a negligible intrinsic charge doping. At the same bias, Fig. 2c also shows emission from XX^0^. In the electron-charged regime (*V* > 0) fluorescence from X^0^, XX^0^ and X^0^_dark_ vanishes, while emission from X^−^_inter_, X^−^_intra_ and XX^−^arises. Around 2 V the X^−^ emission switches to a new peak at ~1.681 eV, likely the next charging state of the trion, X^−−^. This peak was previously assigned to the fine structure of X^−^ in experiments on bare material^10^. Negative bias is the hole-charged regime, where only X^0^ and the positively charged trion X^+^ are visible (refs. ^10,35^). The voltage-dependence of our PL measurements thus clarifies the difference between the two biexciton species: the presence of XX^0^ only at charge neutrality confirms this is the charge-neutral biexciton, and the appearance of XX^−^ only in the electron-charged regime proves it to be the negatively charged biexciton.

We then analyse the correlation between excitation and emission polarisations in the different charging regimes (Fig. 2d). We plot the degree of circular polarisation [DoP = (*I*_co._- *I*_cross._) / (*I*_co._ + *I*_cross._)^4^] where *I*_co._ (*I*_cross._) is the intensity of the circularly polarised light with the same (opposite) helicity in the excitation and detection paths. We refer to the two orthogonal helicities as *σ*^−^ and *σ*^*+*^. At 0 V, XX^0^ has DoP > 80%, while X^0^_dark_ shows no circular polarisation^36,37^, as expected. At 0.8 V, X^−^_inter_ has DoP > 90%, X^−^_intra_ has DoP < 10% and XX^−^ has DoP ~ 55%. The circular polarisation of photons from both XX^0^ and XX^−^ thus implies that dissociation occurs with the recombination of a bright exciton, as a dark exciton would emit linearly polarised light^36,37^. The DoP of XX^−^ is close to the average of the DoP of X^−^_inter_ and X^−^_intra_, suggesting that the recombination mechanisms of both X^−^_inter_ and X^−^_intra_ contribute^38^ to that of XX^−^.

### Behaviour of exciton complexes in magnetic field

**Fig. 3:**
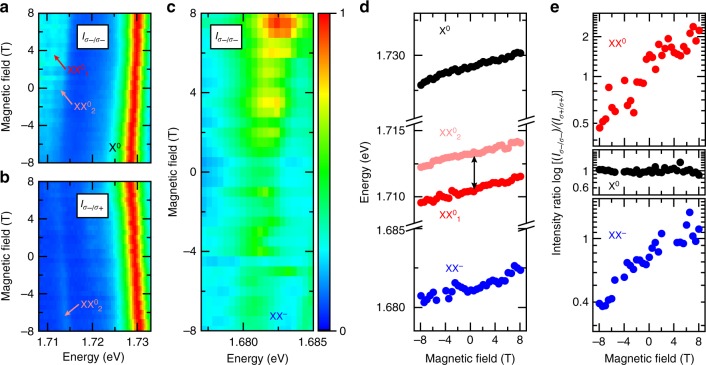
Magnetic field dependence of PL. **a** Magnetic field dependent PL of X^0^ and XX^0^ in circular co-polarised and **b** cross-polarised configurations, for *σ*^−^ excitation. The fine-structure lines are indicated as XX^0^_1_ and XX^0^_2_. The emission of XX^0^ brightens with increasing emission energy. X^0^ is displayed for reference. **c** Magnetic field dependent PL of XX^−^ in a circular co-polarised configuration, for *σ*^−^ excitation. In **a**, **b** and **c** the colour scale is linear. **d** Zeeman shift in the PL spectrum of X^0^ (filled black circles), XX^0^ (filled red and pink circles for the two components of the fine-structure) and XX^−^ (filled blue circles). The double arrow is a scale bar of 2.5 meV. **e** PL intensity ratio of circular co-polarisation with opposite helicity *I*(_*σ-/σ-*_)/*I*(_*σ+/σ+*_) for X^0^, XX^0^_1_ + XX^0^_2_ and XX^−^ as a function of magnetic field

The electrons and holes comprising the biexcitons can occupy multiple combinations of band states. To identify them, we resort to the variation of PL as a function of an out-of-plane magnetic field. Figure 3a shows the *σ*^−^ polarised PL of X^0^ and XX^0^ under co-polarised (*σ*^−^) excitation. We resolve a finite splitting in the XX^0^ emission, with a separation of 2.5 meV between the two peaks labelled XX^0^_1_ and XX^0^_2_ (line-cut spectra at different magnetic fields are shown in Supplementary Fig. 3). Figure 3b shows the *σ*^+^ polarised PL of X^0^ and XX^0^ under cross-polarised (*σ*^−^) excitation. Here, only XX^0^_2_ remains visible, revealing a different DoP for XX^0^_1_ and XX^0^_2_, in analogy to the different DoP between X^−^_inter_ and X^−^_intra_.

The energy of the splitting excludes one of the peaks to be a phonon replica, and the two peaks reveal different DoP, thus we assign this doublet to fine structure introduced by exchange interaction, in analogy to the case of the splitting between X^−^_inter_ and X^−^_intra_^39^. This experimental observation of the XX^0^ fine structure will set a reference for further computational studies, which otherwise suffer from limitations due to the complex treatment of the exchange interaction. Additionally, the PL intensity of XX^0^ emission increases when it shifts to higher energies, in contrast to that of X^0^. We observe the same behaviour for XX^−^ in Fig. 3c, where the co-polarised PL from the recombination of the quasi-particle also shows valley-dependent Zeeman shift.

In Fig. 3d we plot the energies of X^0^, XX^0^_1_, XX^0^_2_ and XX^−^ as a function of magnetic field. For each multi-exciton species, we calculate the Landé factor *g*, defined as *ΔE* *=* *gμ*_B_*B*, where *ΔE* = *E*_*σ+*_−*E*_*σ−*_ is the difference in the emission energy of excitons in opposite valleys, *μ*_B_ = *eħ/2m*_*e*_ = 58 μeV T^−1^ is the Bohr magneton and *B* is the magnetic field. We derive *g* ~ -4.44 ± 0.12 for X^0^ consistent with previous observations^40^, ~ -4.10 ± 0.15 for XX^0^ and ~ -3.86 ± 0.17 for XX^−^. We note that these values do not represent the total g factor of the multi-particle states, but rather belong to their optically active components.

The emission intensities of XX^0^ and XX^−^ change dramatically with magnetic field, being stronger when shifted to higher energy. Figure 3e displays the *I*_*σ-/σ-*_*/I*_*σ+/σ+*_ ratio as a function of magnetic field for XX^0^_1_ + XX^0^_2_ and XX^−^. For comparison, we also include *I*_*σ-/σ-*_*/I*_*σ+/σ+*_ for X^0^. At zero magnetic field *I*_*σ-/σ-*_*/I*_*σ+/σ+*_ is ~1 for all peaks, i.e., the two valleys have the same exciton population. When magnetic field is applied, *I*_*σ-/σ-*_*/I*_*σ+/σ+*_ remains unaffected for X^0^. This can be explained by X^0^ in each valley recombining before reaching thermal equilibrium. In stark contrast, XX^0^ and XX^−^ display strongly anti-symmetric magnetic-field dependence: for increasing magnetic field, the lower-energy transition is weaker.

**Fig. 4:**
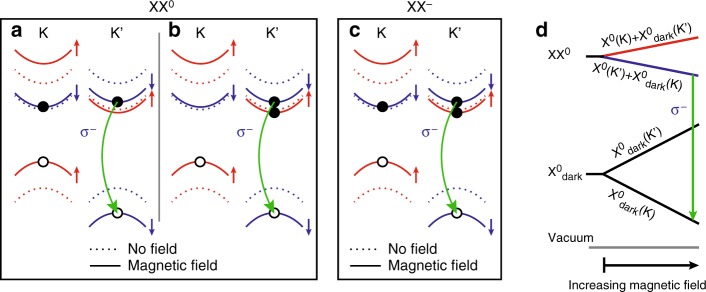
Composition of biexciton species with applied magnetic field. **a, b, c** Single-particle picture of the internal structure of (**a, b**) XX^0^ and (**c**) XX^−^ for *B* > 0. The eigenstates shift inequivalently in K and K′ (dashed curves indicate no magnetic field, solid curves indicate applied magnetic field, red and blue colours indicate opposite spin). XX^0^ comprises a bright exciton with highest radiative energy and a dark exciton with the electron (**a**) inter- or (**b**) intra-valley with the bright exciton. **d** Many-body picture of the magnetic field effect on XX^0^, comprising a bright and a dark exciton. Applying a magnetic field shifts the energy of the dark exciton more than that of XX^0^ due to the higher *g* of the former. This results in the dissociation of the biexciton in the form XX^0^ → X^0^_dark_ + γ(*σ*^−^), where γ(*σ*^−^) is a photon with *σ*^−^ helicity

We can understand the complex behaviour of the magnetic-field dependent PL through the single-particle picture of the energy bands. Figure 4a, b, c illustrates the effect of *B* > 0 on the band structure of 1L-WSe_2_ around the K and K′ points_,_ considering the contribution of the spin, valley and atomic orbital magnetic moments. The 1L-WSe_2_ bandgap decreases (increases) in the K (K′) valley as the energies of both hole and electron experience the same spin upshift (downshift), while the hole experiences a larger orbital upshift (downshift)^40,41^ with respect to the electron. Further, the contribution from the valley magnetic moment results in an additional upshift (downshift) of all bands in the K (K′) valley^40^. The applied magnetic field induces unequal bright exciton populations in the two valleys (Fig. 3e). This excludes the possibility that XX^0^ (Fig. 4a, b) may be formed by two bright or two dark excitons, as both cases would result in equally intense radiative recombination from both K and K′ at all magnetic fields. XX^0^ is therefore a combination of a bright and a dark exciton. Under positive magnetic field, the bright exciton component of XX^0^ occupies the higher-radiative energy transition in the K′ valley (Fig. 3a, b, e) due to thermalisation of the photogenerated electrons. We expect this to be allowed by a longer lifetime of XX^0^ compared to X^0^, in analogy to XX^−^, where the lifetime was measured to be ~2–100 times longer than single excitons^20,25^, and also exhibiting similar polarisation properties. In parallel, the electron of the dark component of XX^0^ can be either in the opposite (Fig. 4a) or in the same (Fig. 4b) valley as the bright exciton component, yielding an energy shift between these two configurations, which is the origin of the fine-structure of XX^0^ observed in Fig. 3d.

Figure 4c illustrates the single-particle configuration of XX^−^. As for XX^0^, the combination of two bright excitons is excluded due to different recombination intensities in K and K′. From the similar *g* of XX^0^ and XX^−^, we can understand this five-particle complex as a bound state of a bright exciton with a dark trion, or a bright trion with a dark exciton. Both configurations would show inequivalent valley population as for XX^0^ in Fig. 3e.

Figure 4d is a qualitative many-body picture for XX^0^ formed by a bright and a dark exciton component in opposite valleys under magnetic field. As its total Zeeman splitting depends on both the bright and the dark component, XX^0^ splits with a reversed energy order compared to its bright exciton component and dissociates into a dark exciton and a photon due to the dark exciton having larger *g* than X^0^ with opposite sign^42^. The distribution of biexciton states follows the case near thermal equilibrium, which is the reason behind the inequivalent circularly co-polarised emission intensity under *σ*^+^ or *σ*^−^, as shown in Fig. 3.

## Discussion

We have discovered the quinton, the five-particle negatively charged biexciton in 1L-WSe_2_, unambiguously, as well as the neutral biexciton and its fine structure. Immediate next steps include the unequivocal verification of the X^−−^ state and the identification of bound states within the lower-energy peaks. A complete understanding of multi-exciton complexes is key to study coherent many-body phenomena, such as condensation^7,8^ and superfluidity^9^. Further, the ability to access and manipulate biexciton complexes in TMD-based heterostructures offers new routes towards probing other fundamental excitations in this system and the interplay between free and localised excitons. Extending our findings to the quantum confined regime will open new capabilities for cascaded emission of entangled photons and spin-multiphoton interfaces.

## Methods

### Sample fabrication and room-temperature characterisation

Bulk WSe_2_ crystals are prepared by the flux zone growth method (see Supplementary Note 1). Bulk hBN crystals are grown by the temperature-gradient method under high pressure and high temperature. Graphite is sourced from NGS Naturgrafit. All bulk crystals are exfoliated by micromechanical cleavage^43^ on Si/SiO_2_ (oxide thickness 285 nm). 1L- and FL-samples are identified by optical contrast^44^. Selected crystals are assembled within ~5 h into LMHs via dry-transfer^13,34^. The LMH sample used for power-dependent and magnetic field-dependent PL measurements is formed, from top to bottom, of ML-hBN flakes (~5 nm thick as determined by optical contrast), 1L-WSe_2_, and a second ML-hBN flake (~10 nm thick as determined by optical contrast) and FLG (~5 layers thick as determined by optical contrast). That used for voltage-dependent measurements is prepared in a similar way, but the top ML-hBN does not fully cover the 1L-WSe_2_ to allow for Cr/Au (5/50 nm) electrodes to directly contact it. The second electrode contacts FLG. The electrodes are patterned by e-beam lithography followed by lift-off. The ML-hBN thickness is chosen to isolate the 1L-WSe_2_ from the environment, smoothen the roughness of SiO_2_, shield the charge-traps of the substrate and avoid tunnelling between FLG and 1L-WSe_2_, while not compromising the optical contrast under the optical microscope. Raman spectroscopy (Supplementary Fig. 1) and PL (Supplementary Fig. 2) are performed on the bulk crystals and after the assembly of LMH to characterise the starting material and confirm the 1L-WSe_2_ thickness^45–47^. Raman and PL spectra are acquired at room temperature using a Horiba LabRam Evolution (spectral resolution ~0.3 cm^−1^) at 514.5 nm. See Supplementary Note 2 for details on the room-temperature optical characterisation.

### Optical measurements at 4 K

Power dependent and gate-controlled measurements are performed in a variable-temperature Helium flow cryostat (Oxford Instruments Microstat HiRes2) with a home-built confocal microscope at a nominal temperature of 4.2 K. The magneto-optical measurements are performed in a close-cycle bath cryostat (Attocube Attodry 1000) equipped with a superconducting magnet (maximum out-of-plane magnetic field 8 T) at a nominal sample temperature of 3.8 K. In the main text we refer to measurements at 4 K as an average of these two nominal temperatures.

### Theoretical calculations

We use Mott–Wannier model and quantum Monte Carlo (QMC) as implemented in CASINO^48^ to calculate the energies of X^0^, XX^0^ and XX^−^in ML-WSe_2_^29^. The full photoemission spectra of ML-WSe_2_ in vacuum are reported in ref. ^29^. To consider the effect of the dielectric screening provided by hBN, we use the experimental value of the binding energy of XX^0^ and use Eq. (48) of ref. ^29^ to derive the screening parameter r* which is 54 Å. We use the many-body GW electron and hole effective masses as 0.29*m*_0_ and 0.34*m*_0_^49^, respectively, where *m*_0_ is the bare electron mass. We calculate the binding energy of XX^−^ by subtracting the total energy of the exciton and trion from the total energy of XX^−^.

### Data availability

The datasets generated and analysed during the current study are available from the corresponding author on reasonable request.

## Electronic supplementary material

Supplementary Information
