# Supplementary material for: Charge-tuneable biexciton complexes in monolayer WSe2
Source: Nat Commun. 2018 Sep 13;9:3721. doi: 10.1038/s41467-018-05632-4 (PMC6137137; doi:10.1038/s41467-018-05632-4)
Supplement: Supplementary file 1 — Supplementary Information [file 41467_2018_5632_MOESM1_ESM.pdf]

Supplementary Information for

"Charge-tuneable biexciton complexes in monolayer WSe_2"

Matteo Barbone[1,2†], Alejandro R.-P. Montblanch[1†], Dhiren M. Kara[1], Carmen Palacios-Berraquero[1], Alisson R. Cadore[2], Domenico De Fazio[2], Benjamin Pingault[1], Elaheh Mostaani[2], Han Li[3], Bin Chen[3], Kenji Watanabe[4], Takashi Taniguchi[4], Sefaattin Tongay[3], Gang Wang[2], Andrea C. Ferrari[2]*, Mete Atatüre[1]*

Affiliations:

[1]*Cavendish Laboratory, University of Cambridge, JJ Thomson Ave., Cambridge CB3 0HE, UK*

[2]*Cambridge Graphene Centre, University of Cambridge, Cambridge CB3 0FA, UK*

[3]*School for Engineering of Matter, Transport and Energy, Arizona State University, Tempe, AZ 85287, USA*

[4]*Advanced Materials Laboratory, National Institute for Materials Science, Tsukuba, Ibaraki 305-0034, Japan*

[†]these authors contributed equally to this work

Supplementary Note 1: Growth of bulk WSe_2

W and Se pellets with ultra-high purity (Puratronic 99.9999% or higher) are sealed in a quartz ampoule at 10^{-6} Torr pressure. We adopt a two-step growth approach: in the first step, W and Se pellets are sealed at stoichiometric ratios (~500 mg total weight) in a quartz ampoule. The pressure is kept at 10^{-6} Torr and the temperature at 1050 ^{0}C, slightly below the actual growth temperature of 1065 ^{0}C. Before sealing in vacuum, T~300 ^{o}C is applied to remove any residual molecules adsorbed on the precursors as well as on the quartz reactor walls. After sealing, the ampoule is at 1050 ^{o}C for 1 week to produce polycrystalline WSe_2 powders. The WSe_2 powders are checked with scanning electron microscope-energy dispersive spectroscopy (SEM-EDS) to determine the amount of Se vacancies. In the second step, ~5 mg extra Se is sealed with the WSe_2 powders in a quartz ampoule at 10^{-6} Torr. The excess Se is added to help reduce potential Se vacancies. The sealed ampoule is then heated to 1090 ^{o}C over 3 days. The side without precursors is cooled by 25 ^{o}C to 1065 ^{o}C within an hour to create the thermodynamic flux to initiate the growth[1]. This process typically leads to crystalline WSe_2 flakes that are a few mm in size.

Supplementary Note 2: Room-temperature optical characterization

The LMHs are characterized by Raman spectroscopy and PL using a Horiba LabRAM Evolution spectrometer equipped with a 100x objective (N.A. 0.6). Supplementary Figure 1 (a,b) plot the spectrum of bulk (red curve) and 1L-WSe_2 (magenta curve). In 1L-WSe_2, the E_{2g}^1 and A_{1g} modes[2] are merged in a single band at ~250 cm^{-1} and the shear mode C, due to relative motion of atoms in adjacent planes, is not present[3]. Bulk WSe_2 shows split E_{2g}^1 and A_{1g} modes at ~249 cm^{-1} and at ~251 cm^{-1} (Supplementary Reference 2). The C mode is detected at ~24 cm^{-1} (Supplementary Reference 3). Supplementary Figure 1c,d plots the Raman spectra of the resulting layered material heterostructures, corresponding to Fig. 2 in the main text. The black curve in Supplementary Figure 1c,d is the spectrum of FLG on SiO_2/Si with the characteristic G peak at ~1580 cm^{-1} and the 2D band at ~2720 cm^{-1} (Supplementary Reference 4). The green curve is the spectrum of hBN on FLG. The peak at ~1366 cm^{-1} belongs to the E_{2g} mode of hBN[5,6]. The blue curve is the spectrum of ML-hBN/1L-WSe_2/ML-hBN/FLG. This shows that all the features of the forming individual layers are preserved.

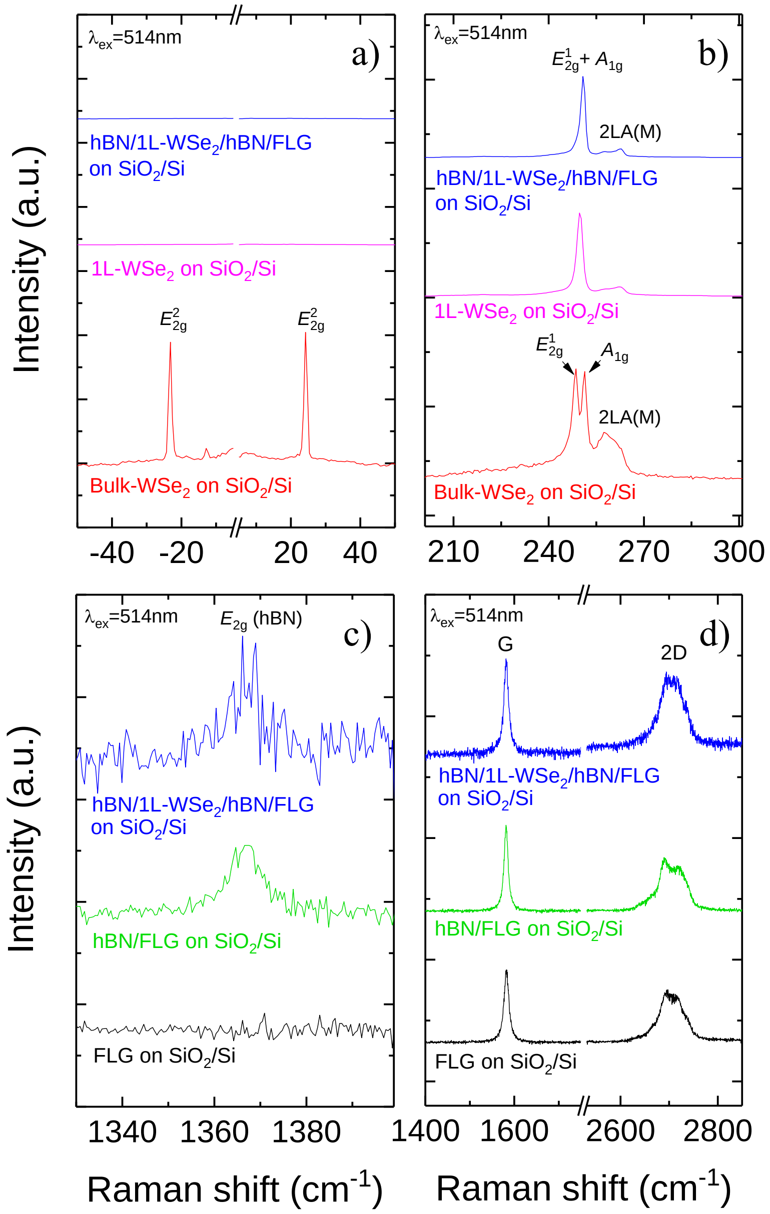

Supplementary Figure 1: Raman spectra of layered material heterostructure. a Low frequency and **b** high frequency Raman spectra of Bulk WSe_2 (red), 1L-WSe_2 (magenta) on Si/SiO_2 and ML-hBN/1L-WSe_2/ML-hBN/FLG (blue). **c** E_{2g} peak of hBN and D peak spectral regions and d) G and 2D peaks of FLG spectral region in FLG on SiO_2 (black); ML-hBN/FLG (green) on Si/SiO_2 and ML-hBN/1L-WSe_2/ML-hBN/FLG (blue).

PL (Supplementary Figure 2) is collected to confirm the monolayer nature of the WSe_2 crystal. The peak at ~750 nm corresponds to the neutral X^0 exciton[2]. Supplementary Figure 2 compares the PL spectrum of 1L-WSe_2 on SiO_2 (magenta) and in the layered material heterostructure (blue) with that of bulk WSe_2 (red) keeping the same measurement conditions. The PL intensity

drastically decreases in bulk WSe_2 with respect to 1L-WSe_2 due to a direct-to-indirect bandgap transition[7]. The shape of the 1L-WSe_2 PL is preserved in the layered material heterostructure.

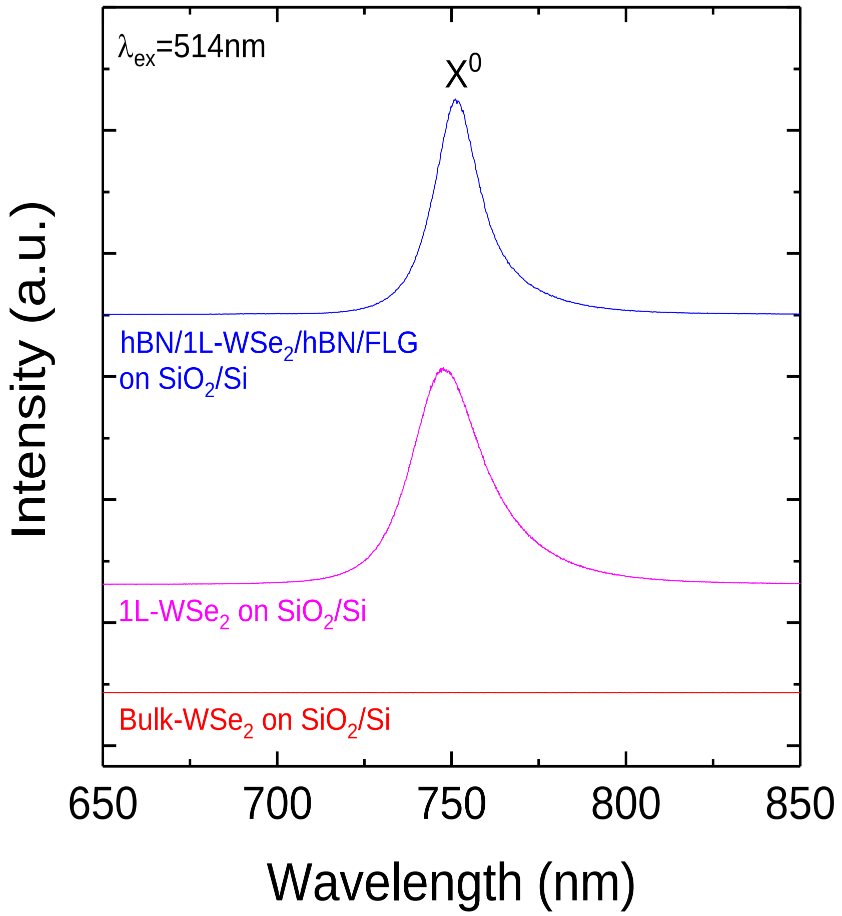

Supplementary Figure 2: PL spectra at RT. Blue curve shows the X^0 exciton at ~750 nm, which confirms the presence of 1L-WSe_2. The red curve is the PL spectrum of bulk WSe_2 on SiO_2/Si measured keeping the same laser power as that of 1L-WSe_2 (magenta curve). The blue curve is the PL spectrum of the layered material heterostructure.

Supplementary Figure 3: Spectra of the fine structure of XX^0

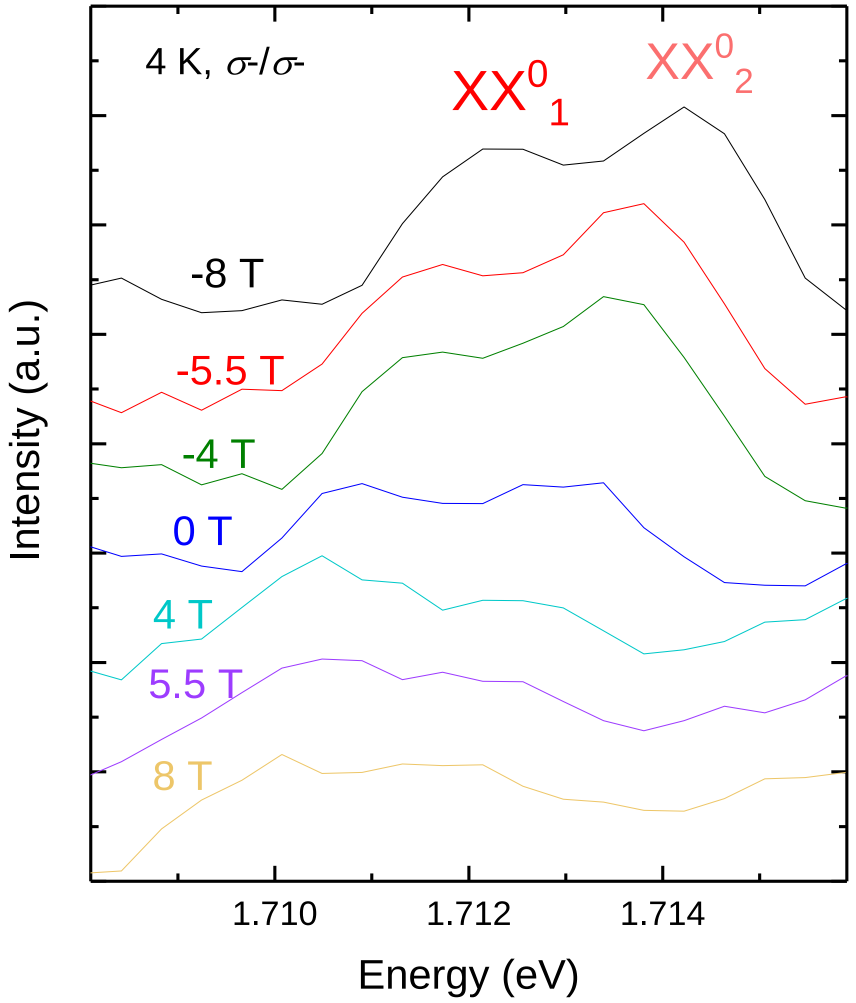

Supplementary Figure 3: Line-cut spectra of XX^0 as function of applied magnetic field. Spectra are taken at 4 K and different magnetic fields with a circular co-polarized scheme and σ^- helicity, showing the double peak formed by XX^0_1 and XX^0_2, after linear background subtraction.

Supplementary References:

1. Zhang X. *et al.*, Flux method growth of bulk MoS_2 single crystals and their application as a saturable absorber. *Cryst. Eng. Commun*. **17**, 4026-4032 (2015).

2. Tonndorf P. *et al.*, Photoluminescence emission and Raman response of monolayer MoS_2, $MoSe_2$, and WSe_2. *Opt. Express* **21**, 4908 (2013).

3. Tan P. H. *et al.* The shear mode of multilayer graphene. *Nat. Mater.* **11**, 294 (2012).

4. Ferrari A. C. *et al.* Raman spectrum of graphene and graphene layers. *Phys. Rev. Lett.* **97**, 187401 (2006).

5. Reich S. *et al.* Resonant Raman scattering in cubic and hexagonal boron nitride. *Phys. Rev. B* **71**, 205201 (2005).

6. Arenal R. *et al.* Raman spectroscopy of single-wall boron nitride nanotubes. *Nano Lett.* **6**, 1812 (2006).

7. Mak K. F. *et al.* Atomically thin MoS_2: a new direct-gap semiconductor. *Phys. Rev. Lett.* **105**, 136805 (2010).
